# Macromolecular Interactions Control Structural and Thermal Properties of Regenerated Tri-Component Blended Films

**DOI:** 10.3390/ijms17121989

**Published:** 2016-11-28

**Authors:** Ashley Lewis, Joshua C. Waters, John Stanton, Joseph Hess, David Salas-de la Cruz

**Affiliations:** 1 Department of Biology, Rutgers University-Camden, 315 Penn Street, Camden, NJ 08102, USA; aml304@scarletmail.rutgers.edu (A.L.); jcwmol@yahoo.com (J.C.W.); 2 Department of Chemistry, Rutgers University-Camden, 315 Penn Street, Camden, NJ 08102, USA; jstant14@scarletmail.rutgers.edu (J.S.); jmh433@scarletmail.rutgers.edu (J.H.); 3 Center for Computational and Integrative Biology, Rutgers University-Camden, 315 Penn Street, Camden, NJ 08102, USA

**Keywords:** biomaterials, ionic liquids, morphology, lignin, self-assembly, microfibril, X-ray scattering, lignocellulose

## Abstract

With a growing need for sustainable resources research has become highly interested in investigating the structure and physical properties of biomaterials composed of natural macromolecules. In this study, we assessed the structural, morphological, and thermal properties of blended, regenerated films comprised of cellulose, lignin, and hemicellulose (xylan) using the ionic liquid 1-allyl-3-methylimidazolium chloride (AMIMCl). Attenuated total reflectance Fourier transform infrared (ATR-FTIR) analysis, scanning electron microscopy (SEM), atomic force microscopy (AFM), X-ray scattering, and thermogravimetric analysis (TGA) were used to qualitatively and quantitatively measure bonding interactions, morphology, and thermal stability of the regenerated films. The results demonstrated that the regenerated films’ structural, morphological, and thermal character changed as a function of lignin-xylan concentration. The decomposition temperature rose according to an increase in lignin content and the surface topography of the regenerated films changed from fibrous to spherical patterns. This suggests that lignin-xylan concentration alters the self-assembly of lignin and the cellulose microfibril development. X-ray scattering confirms the extent of the morphological and molecular changes. Our data reveals that the inter- and intra-molecular interactions with the cellulose crystalline domains, along with the amount of disorder in the system, control the microfibril dimensional characteristics, lignin self-assembly, and possibly the overall material′s structural and thermal properties.

## 1. Introduction

With growing environmental and economic concerns, current scientific research is looking towards renewable raw materials for manufacturing and technology [1,2,3]. Due to extreme natural abundance, lignocellulose has received interest as a source of biomass that is both sustainable and cost effective [4,5,6,7,8]. Cellulose, the most heavily explored component of lignocellulose, has been used in areas such as biomedical research, fuel production, and 3D printing [6,9,10,11,12]. This linear polysaccharide represents the main component of plant cell walls and contains both crystalline and amorphous regions. It is one of the most abundant materials in the world, found in nature as cellulose I (Iα or Iβ) [13]. As a polymer of glucose with β(1–4) glycosidic bonds, cellulose is capable of forming strong intramolecular and intermolecular hydrogen bonding mainly via its –OH functional groups [13,14]. In the lignocellulose structure itself, cellulose is embedded in a matrix of hemicelluloses [15], with this cellulose-hemicellulose network further encapsulated by an outer lignin layer [16]. Hemicellulose, a mixture of various complex polysaccharides, has a diverse composition across plant species. Most consistently, hemicelluloses include significant amounts of xylan, the glucan backbone of 1,4-d-xylopyranose that possesses branched chains of 4-oxymethylglucuronic acid [17]. The last main component of lignocellulose, lignin, is a highly-crosslinked, aromatic structure with a number of methoxy side groups. The combination of aromaticity and electron-donating groups serve to increase hydrophobicity of the secondary cell wall resulting in enhancements of material physical properties [16].

This biopolymer network is maintained mostly through extensive hydrogen bonding between all three components [17]. However, covalent bonding amongst the components does exist, such as the ester bonding between lignin and hemicelluloses [18,19] and ether bonding between cellulose and lignin [20]. Amongst plant species, the various combinations of inter- and intra-molecular bonding within lignocellulose contributes to the diversity seen in the strength and function of plant cell walls. Consequently, plant species will have distinct lignocelluloses bonding phenotypes that manifest different physical properties, such as cell wall strength [16]. For instance, plant species with increased lignin content, like wood species, contain an increase in rigidity to their cell walls [21].

In most industries and areas of study, isolated cellulose has been the primary focus for new technologies. One particular advancement in the application of cellulose is the formation of regenerated cellulose films, which entails the physical dissolution and regeneration of cellulose into films using a number of potential solvents [22]. Regenerated cellulose films have a wide variety of applications across many fields, such as the packaging [23], fuel [24], textiles [25], and biomedical fields [9,12,26]. In recent years, some of the most popular cellulose solvents have been ionic liquids (ILs), which have been a shown to powerfully dissolve cellulose for film synthesis without breaking the cellulose molecular weight [7,27,28,29,30,31,32,33]. ILs are a popular solvent choice due to their unique properties of chemical and thermal stability, non-flammability, and immeasurable low vapor pressure [30]. These characteristics of solvents are essential, as they are the driving force for the dissolution of the polysaccharide. A poor solvent will significantly affect the miscibility of polysaccharide-based film, reducing its mechanical properties, and could destroy the original molecular weight of natural macromolecules [34,35]. Although this abundance of information about cellulose’s properties and application is necessary for biomaterial research, it is important to remember the earlier discussion which elucidated that natural cellulose is only one component in a larger network of possible natural biopolymers. Due to additional interactions, new properties may emerge when two or three natural macromolecules are blended together, properties which are not observed in isolated cellulose studies. For instance, unblended cellulose I, upon coagulation, would be dissembled to cellulose II in various solvents. Yet it is unknown whether this dissolution characteristic also applies when cellulose is blended with other macromolecules. For example, it has been reported that the structure of regenerated cellulose also occurs in amorphous and intermediated forms [36,37,38,39]. It has been suggested that larger molecules or certain types of ionic liquids could have a direct effect on the recrystallization of cellulose to intermediated structures by imposing constraints or crystallization disruptions [36,37,38]. As discussed, the exploration of regenerated cellulose films has been extensively investigated [25,28,29,40,41], but the regeneration of all lignocelluloses components together—lignin, cellulose, and hemicellulose (xylan), and the details pertaining to the structural characteristics of lignocellulose-regenerated films—has yet to be fully explored. Therefore, our motivation for this study is to characterize a novel biomaterial by blending all three of lignocellulose’s biopolymers together. Our goal is to illuminate the macromolecular interfacial interaction effect on the cellulose crystallinity, lignin self-assembly, and other relevant features, possibly revealing new and tunable properties.

Considering the array of information that can be gained from the investigation of blended biopolymers, our group performed a series of characterization experiments on regenerated films containing lignin, cellulose, and xylan. Our objective of this study aims to observe the structural, thermal and morphological properties that occur when the amounts of lignin and xylan were altered relative to a constant concentration of cellulose. To achieve this, regenerated films were generated using 1-allyl-3-methylimidazolium chloride ionic liquid and coagulated with water. The films were analyzed using Attenuated total reflectance Fourier transform infrared (ATR-FTIR) analysis, scanning electron microscopy (SEM), atomic force microscopy (AFM), X-ray scattering, and these various analyses were used to elucidate if the structure and morphology of the films changed as a function of lignin-xylan concentration. In addition, using thermogravimetric analysis (TGA), the thermal stability was evaluated by looking at both the intra- and inter-molecular levels to see if thermal properties correlated to changes in component concentration. Our results revealed significant and decisive influence cause by the lignin-xylan concentrations upon the properties of regenerated lignocelluloses films, especially information relating to the cellulose microfibrils and lignin self-assembly.

## 2. Results and Discussion

### 2.1. ATR-FTIR Analysis

In this study, we assessed the properties of blended, regenerated films comprised of varying lignocellulose component concentrations using the ionic liquid 1-allyl-3-methylimidazolium chloride (AMIMCl). Table 1 details the composition breakdown for the cellulose, lignin, and xylan proportions for each film. These concentration ranges were chosen in order to mimic the proportions found in natural plant species, with cellulose levels typically around 45% and lignin concentrations no greater than 40% [16,21]. The upper and lower ranges for lignin and hemicelluloses (xylan) were chosen based on the extremes present in various plants, with the lowest concentrations approximately 17% and the highest approximately 38%. With a relatively consistent concentration of cellulose, the concentrations of hemicelluloses and lignin tend to be inversed in nature. Therefore, seeing opposing extremes for lignin and hemicelluloses will help identify which characteristics are related to each particular component. The 27.5% film was generated to see how equal concentrations of lignin and xylan interacted in regenerated film form. Experiment 1 was conducted as a control which served a point of comparison to past studies, which utilized only cellulose in regenerated films. Experiments 2–4 varied proportions of xylan and lignin with a constant concentration of cellulose. The constant cellulose concentration in Experiments 2–4 allowed for increased certainty that the changes observed were due to the alterations of xylan and lignin exclusively. For the purposes of this paper, the regenerated film containing pure cellulose will be referenced as “100% cellulose film” while the pure, raw cellulose material will be referenced as “microcrystalline cellulose”.

**Figure 1:**
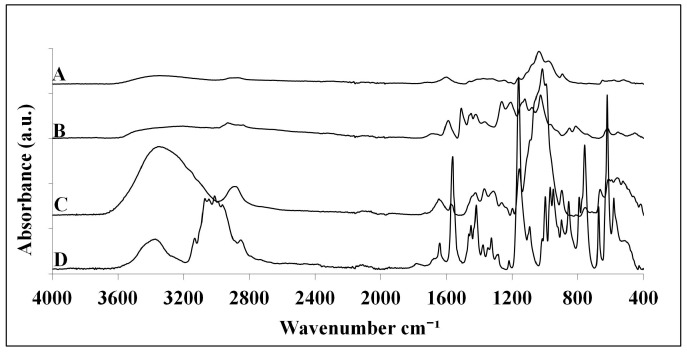
Attenuated total reflectance Fourier transform infrared (ATR-FTIR) spectra of pure material components. (**A**) Xylan; (**B**) lignin; (**C**) microscrystalline cellulose; and (**D**) AMIMCl.

As Figure 1 details, the IR absorbance intensities for the pure components of lignocellulose and AMIMCl have their own characteristic spectrum. Although the biomass share some overlapping bands, microcrystalline cellulose has the most pronounced peaks at the –OH (3000 cm^−1^) and C–O (1060 cm^−1^) groups. Alternatively, xylan has a stronger peak at 1600 cm^−1^, which is indicative of C=O stretching. Lignin, on the other hand, shows the most unique IR expression in the fingerprint (700–1800 cm^−1^), with especially strong peaks at the methoxy (1450 cm^−1^) and aromatic (1630 cm^−1^) groups. In the spectrum for AMIMCl, there are pronounced peaks at 3050, 2965, 1570, and 1165 cm^−1^, with these IR regions previously being characteristic of AMIMCl [42]. Since the ATR-FTIR spectra of AMIMCl and lignocellulose component samples used in this study corroborate past results [42,43], they can reliably be used as markers for analysis of the spectra for Experiments 1–4.

**Figure 2:**
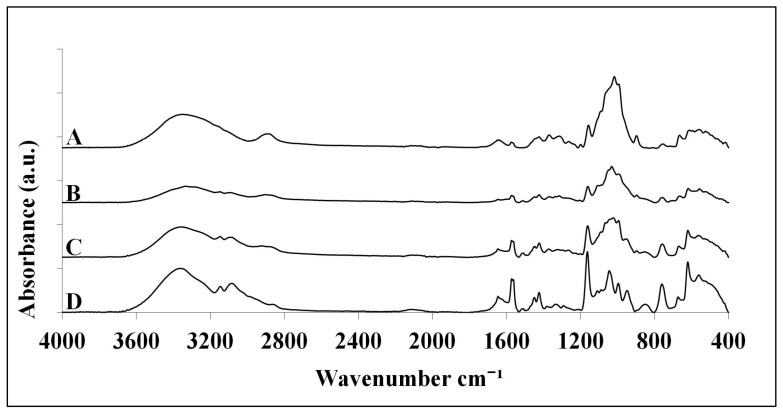
Full length ATR-FTIR spectra as a function of lignin content: (**A**) 100% cellulose film; (**B**) 17% lignin film; (**C**) 27.5% lignin film; and (**D**) 38% Lignin Film.

As can be seen from the IR data in Figure 2, the spectra of the blended films correspond in similarity to the predominant lignocelluloses component to which they are comprised. As expected, the 100% cellulose film matches with the unmixed microcrystalline cellulose spectra. Unlike the blended films, the hydroxyl and carbon-oxygen peaks of the 100% cellulose film appear strong and unadulterated. This is not the case, though, in Experiment 2 (17% lignin), which has a 38% xylan concentration and very diminished –OH (3000 cm^−1^) and C–O (1060 cm^−1^) peaks. The absorbance pattern of this film appears much closer to the xylan absorbance bands at these key cellulose peaks, which follows from the fact that xylan, in the 17% lignin film, is indeed at its highest concentration. Experiment 4, which contains the highest lignin concentration of 38%, manifest the complex pattern of methoxyl and carbon-oxygen peaks between 1600 and 900 cm^−1^ that is characteristic of pure lignin (Figure 1B). When compared to the 100% cellulose film (Figure 2A) for the fingerprint region, the lignin IR identity in the 38% lignin film seems to predominate over the cellulose IR peaks more than in the other, less lignin concentrated films. With these results, it can be confirmed that lignin was present in high concentrations.

Additionally, as lignin concentration is increased, there appears to be more interaction between the film and ionic liquid, as seen by the increase AMIMCl related peak (1165 cm^−1^ for C–H stretching of the imidazolium ring) in Experiment 4. Normally for cellulose-only films, the ionic liquid diffuses into cellulose microfibrils, swelling the structures. The anion of the ionic liquid disrupts hydrogen bonds with the hydroxyl groups of cellulose, interrupting the crystalline hydrogen bonding and causing it to change conformation [44]. The increased amounts of lignin increases intermolecular interaction with the ionic liquid causing possible changes to the inter- and intra-intermolecular bonding with the cellulose and xylan as the ionic liquid diffuses out to regenerate the blended film.

### 2.2. Morphological Analysis

**Figure 3:**
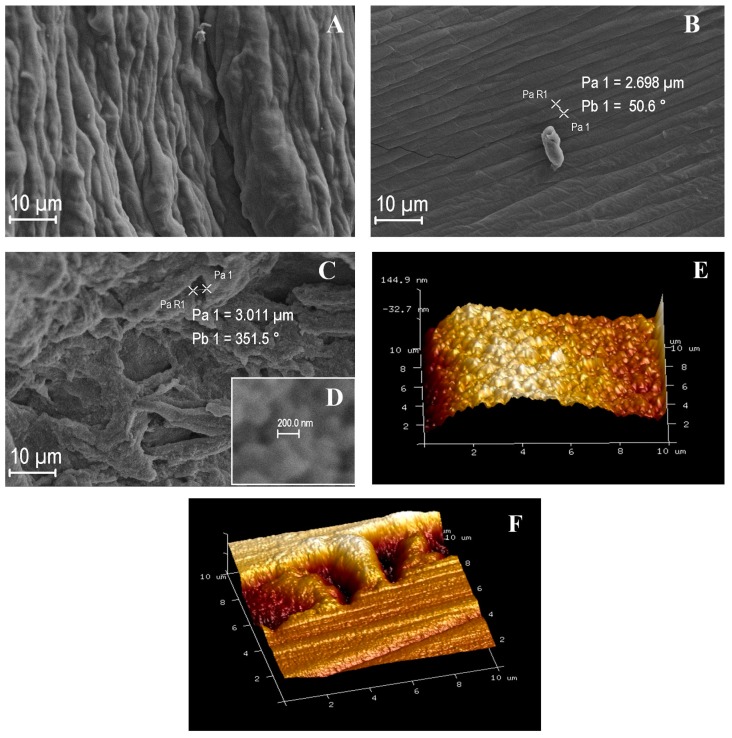
Scanning electron microscopy (SEM) images: (**A**) 17% lignin film; (**B**) 27.5% lignin film; (**C**) 38% lignin; (**D**) 38% lignin at 100,000×; (**E**) atomic force microscopy (AFM) image of 27.5% lignin film; and (**F**) AFM image of 38% lignin film.

These insights gained from IR spectroscopy about blended biopolymer properties are further expanded by the SEM analysis. As can be seen in Figure 3, the SEM images demonstrate marked changes in film morphology according to lignin-xylan proportions. In Figure 3A,B, the films with less lignin (17% and 27.5%) and elevated xylan concentrated films have more fibrous structures on their surfaces. Alternatively, as lignin content is increased in the films (Figure 3C, 38% Lignin), the surface acquires a ridge topology with spherical patterns. The spherical structures on the 38% film can be seen in Figure 3D in greater resolution, measuring at an average size of 250–350 nm.

Since it cannot be determined conclusively within the resolution of SEM whether the lignocellulose clusters are artifacts, two films with starkly contrasting SEM morphologies, 27.5% and 38% lignin films, were analyzed using AFM to precisely characterize these structural differences. The AFM results in Figure 3E reveals that the 27.5% lignin film matches the expected fibrous-like structure. The size of the fibers is calculated to be approximately 4–5 µm, which confirms the findings for SEM that revealed fiber sizes of 3–5 µm, as seen on Figure 3B. Alternatively, the AFM for 38% lignin seen in Figure 3F shows the pronounced ridges of globular structures matching those seen on the SEM Figure 3C, with both averaging approximately 2–4 µm. Considering that the surface alterations were observed not only on the SEM, but also validated using AFM, it can be asserted with reasonable certainty that the morphology changes observed represent changes to the structural interactions of the lignocelluloses components.

### 2.3. X-ray Scattering

**Figure 4:**
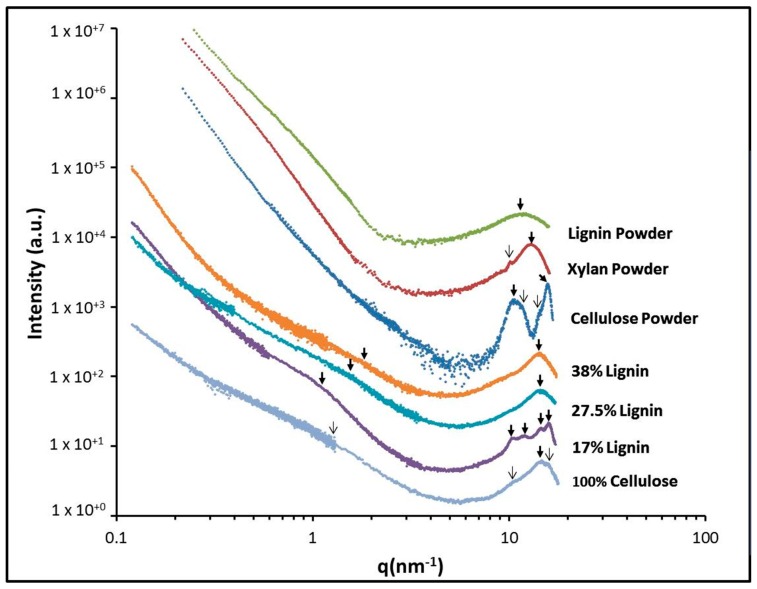
X-ray scattering profiles for the various samples. The intensity is offset to denote the difference between each sample. The arrows indicate the position of each scattering vector peak.

X-ray scattering was used to probe the morphology of the raw materials in pure components and the regenerated films comprised of varying lignocellulose component concentrations over a wide range of scattering vectors. Figure 4 shows the scattering profiles of each sample recorded at room temperature. We initiate our morphological investigation by looking at the distinct spacings that are observed in the pure samples in powder form to understand the differences observed in our regenerated blended films. Two distinct spacings are observed in cellulose and xylan powders, while one is observed in lignin. The spacing in cellulose can be attributed to its crystalline regions (*q*_1_ = 15.75 nm^−1^ and *q*_2_ = 10.61 nm^−1^). Between *q*_1_ and *q*_2_, there are two slightly notable shoulders at *q*_b_ = 11.9 nm^−1^ and *q*_a_ = 14.22 nm^−1^. The d-spacing, for each peak using *d* = 2π/*q* formula, are *d*_1_ of 0.40 nm, *d*_2_ of 0.60 nm, *d*_a_ of 0.44 nm, and *d*_b_ of 0.53 nm, which are related to the monoclinic unit cell of cellulose I_β_ equatorial lattice planes. In support of our data [13], a high-resolution image of the cross-sectioned *Halocynthia papillosa* microcrystal reveals the three d-spacings at 0.60, 0.53, and 0.39 nm, this corresponded to the 1$\bar{1}$0, 110, and 200 reflections and is related to the monoclinic unit cell of cellulose I_β_, respectively [13]. The 0.6 nm index is related to the longer side of the crystal structure, which is always parallel. Helbert and others suggest that the microfibrils could consist of the packing of several sheets of the 0.6-nm lattice. Similar results were reported in Avicel microcrystalline cellulose by other groups [45], with the 0.44 nm related to the layer in the lateral direction or 102 and 012 reflections. For xylan, the spacing can be attributed to amorphous structure from the backbone to backbone correlation of segments (*q*_1_ = 13.11 nm^−1^). We also observe a peculiar spacing at *q*_2_ = 10.11 nm^−1^. The sharpness of this peak indicates possible semi-crystallization of the polymeric structure of the xylan, but it is too small. The d-spacing for each of the peak are *d*_1_ of 0.48 nm and *d*_2_ of 0.62 nm. Other groups have shown similar d-spacing for a typical xylan from beechwood [46]. An amorphous scattering at *q*_a_ = 11.53 nm^−1^ is observed in lignin which is related to the backbone to backbone correlation of segments with a d-spacing of 0.55 nm.

In terms of the regenerated films, the typical sharp crystallization reflections of pure microcrystalline cellulose are not observed. Instead, the regenerated film shows a broad peak at *q*_a_ = 14.57 nm^−1^ which indicates restructuring to an amorphous structure. This is a typical transformation from cellulose I crystalline to amorphous cellulose in the ionic liquid [37]. The d-spacing for this peak is 0.43 nm. This distance is related to the regenerated backbone-to-backbone spacing. The interaction between the various molecules and the ionic liquid changes the conformation of the blended material and, upon coagulation with water, the crystalline domains cannot be formed. It is known that the transformation of cellulose upon coagulation is dominated by type of constituents, temperature, the coagulator, coagulation time, and ionic liquid electronegativity [36]. Upon further inspection, several peak shoulders could be observed: *q*_a_ = 15.79 nm^−1^ and *q*_b_ = 10.61 nm^−1^. These spacings correspond to small structural reflections related to cellulose. Furthermore, a broad peak is seen at the region between the scattering vector (0.33 to 3.10 nm^−1^) by drawing a baseline between the curve. The maximum height of the peak is located at a scattering vector of 1.28 nm^−1^. The d-spacing for the maximum and minimum of this broad peak are 17.0 to 2.41 nm and the average d-spacing is 4.91 nm. This region is related to the average microfibril cross-sectional dimension. Ibbett et al. has reported that microfibril cross-sectional dimension for eucalyptus pulp has a value of 3.6 nm [47], while Ioelovich [48] reported a value of 4.0–4.5 nm for corn cobs. The diameter of microfibrils is known to range from about 2–15 nm where more non-cellulosic polysaccharides may have adhered to the microfibril, such as xylan [49,50].

In continuation, the scattering profile for Experiment 2 (17% lignin) showed five characteristic spacings: *q*_1_ = 15.75 nm^−1^, *q*_2_ = 14.57 nm^−1^, *q*_3_ = 11.95 nm^−1^, *q*_4_ = 10.15 nm^−1^, and *q*_5_ = 1.15 nm^−1^. The d-spacing for each peak is *d*_1_ = 0.40 nm, *d*_2_ = 0.44 nm, *d*_3_ = 0.52 nm, *d*_4_ = 0.62 nm, and *d*_5_ = 5.46 nm. The d-spacings confirm the presence of the various macromolecules in the new regenerated blended system. The sharpness of the peaks at the higher scattering vector is indicative of a semi-crystalline nature of the blended material. In general, unblended cellulose I, upon coagulation, would be disassembled to cellulose II in various solvents; however, recrystallization back to a modified or intermediated structure resembling cellulose I is observed in the blended materials. Recrystallization back to cellulose I has been reported at a temperature of 50 °C and under various experimental conditions [36,37]. Furthermore, it has been reported that constraints or crystallization disruption due to larger molecules or as a function of the type of ionic liquids could have a direct effect on the recrystallization of cellulose intermediated or amorphous structures [36,37,38]. This was also observed in cellulose blended films with lignin and natural macromolecules, where it was reported intermediated structure formed upon blending [38]. In our results, the combination of the tri-components with cellulose causes a type of disruption for the cellulose to recrystallize into an intermediated structure resembling cellulose I. Even though the data clearly exhibits the crystalline regions of cellulose, it also shows the regions related to the xylan and the amorphous lignin.

The scattering profile for the next two blended films shows two distinctive peak at *q*_1_ = 14.45 nm^−1^ and *q*_2_ = 1.64 nm^−1^. The peak at low scattering vector slightly changes for 38% lignin to *q*_a_ = 1.93 nm^−1^. The three d-spacings are *d*_1_ = 0.44 nm, *d*_2_ = 3.83 nm, and *d*_a_ = 3.25 nm. The first peak is related to an amorphous structure while the low scattering vector peak is related to the microfibril diameter in the vertical direction. Herein, the cellulose blend changed from cellulose I crystalline to amorphous structure. There is no evidence of crystallization from these two samples. In observing the lower scattering vector peak for all blended films, we noticed that the breadth of this peak from the 17% lignin to the 38% lignin reduces. Both the peak position and peak intensity clearly demonstrate a relationship between the cellulose and xylan which are known to strongly interact. This is in agreement with the general knowledge that the microfibril diameter can change due to this interaction between cellulose and xylan. Yuan et al. explains that microfibrils are also composed of elementary fibril associated with non-cellulosic polymers like xylan and their average vertical direction dimension are 3.3 nm [44]. As the lignin concentration increases, while reducing the xylan concentration, the peak located at the lower scattering vector region also broaden as seen in the scattering profiles. This means that the microfibril diameter is reduced from 5.46 to 3.25 nm as the percent xylan content is reduced. The data suggests that the cellulose crystalline domain and the amount of disorder in the system control the microfibril dimensional characteristics and possibly its structural and thermal properties. Ibbett et al. pointed out that disorganization of crystals within the fibril (as confirmed by X-ray scattering in our experiments), through disruption, has an increasing effect on its average dimension [47]. In addition, Gillis et al. reported that the parallel glucan chain forms the cellulose microfibril via the crystallization through intra- and inter-hydrogen bonding, which gives it its physicochemical properties [51].

The presented data could enable us to understand the topological changes observed in the SEM images. As the percent lignin increases, the topology changed from a fibrous to a spherical pattern at 38% lignin, suggesting that the molecular interactions with cellulose and xylan are critical for determining whether fibers or spheres form along the surface. It is known that the lignin macromolecule wraps around the microfibril, but when the diameter of the microfibril is reduced, lignin seems to prefer to self-assemble into spherical structures, as shown in the SEM images. Spherical lignin has been observed by others. For example, Zhang et al. observed spherical lignin between 100 and 400 nm in diameter [52]. In that study, Zhang attributed the complexity of the lignin structure, which is a highly-branched polymer consisting of phenylpropane units, with contributing to this aggregation of lignin. Perhaps, interplay between the various intermolecular forces in the microfibrils could result in the intensification of the spheres. We noticed than the diameter of the sphere observed in our SEM data (250–350 nm) is in agreement with Zhang (see Figure 3D). The lower amount of xylan may have contributed to a reduction in the microfibril diameter, which resulted in lignin preferring to self-assemble, forming into spherical structures.

**Scheme 1:**
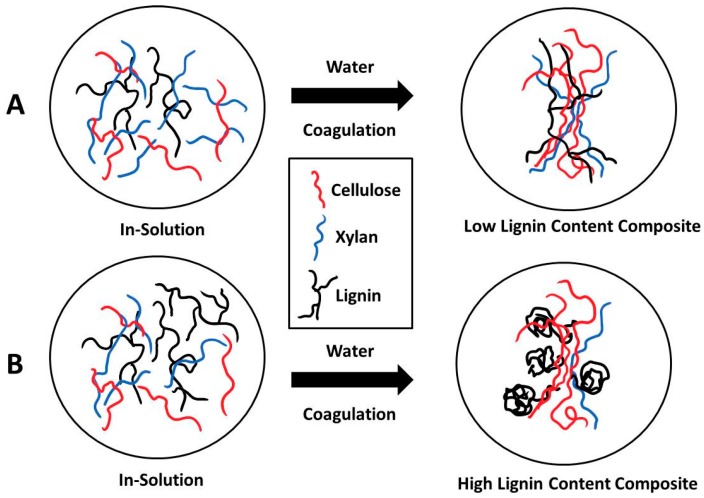
Schematic representation of tri-component random coils in solution and their evolution to solids films at (**A**) low lignin content; and (**B**) high lignin content. Lignin self-assembled spherically at a high lignin content.

From X-ray scattering we learned that the intermolecular forces between xylan and cellulose seem to control the size of microfibrils, which dictates the final morphology of the blended films. Here, we suggest a mechanism similar to Scheme 1. When xylan concentrations are elevated while the lignin content is low, the ordered microfibrils may allow the holocellulose (xylan and cellulose) structures to associate with lignin (see Scheme 1A). In this mechanism, the cellulose and xylan associate to create a larger microfibril diameter, which allows the branched lignin groups to interact along surface. These branches of lignin wrap around the holocellulose microfibril complex in such a way that it provides structural support which manifests as an organized fibrous morphology. Now, when the xylan content is low and the lignin content is high, the microfibril dimension is reduced and the lignin will be unable to wrap around the cellulose, thus preferring to self-assemble due to the lack of anchoring areas (see Scheme 1B). To gain further evidence for this proposed mechanism, let us look at how these changes affect the thermal properties of our system.

### 2.4. Thermal Analysis

**Figure 5:**
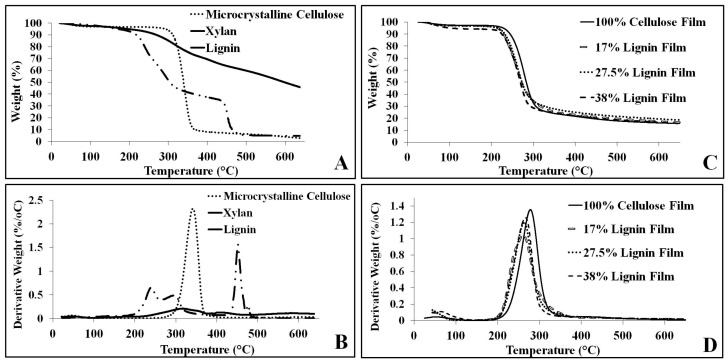
TGA percent weight decomposition analysis for (**A**) pure lignocellulose components; and (**C**) blended films; and TGA derivative percent weight decomposition analysis for (**B**) pure lignocellulose components and (**D**) blended films.

The thermal analysis of the pure material components (Figure 5A,B) and regenerated films (Figure 5C,D) depict similar trends of changes according to lignin-xylan composition. The transformation from crystalline to amorphous structure in cellulose is evident by a large decrease in temperature for onset of decomposition of the 100% cellulose film (*T*_Onset_ = 249.8 °C) compared to the microcrystalline cellulose material (*T*_Onset_ = 341.1 °C). Quantitative data is shown in Table 2. As for lignin, pure lignin degrades slowly from 250–500 °C, which should lead to an increase in the temperature range for decomposition. This characteristic can be seen in the widening of the derivative peak in Figure 5A,B. Although this is observed with the first addition of lignin in the experimental conditions (17% lignin), further increases in the proportion of lignin in the films lead to a narrowing of the decomposition range trending toward the range of the 100% cellulose film. This suggests that there is a distinct intermolecular bonding pattern when lignin is introduced in low concentrations.

In terms of the influence of xylan concentration, the presence of a slight shoulder in the derivative peak for the 17% lignin film can be attributed to its high xylan concentration (38%). Xylan exhibits a unique, two-step decomposition over the range of 200–400 °C. The shoulder seen with the 17% film may be an artifact of the early onset temperature for decomposition of xylan prior to the volatilization of the cellulose component of the film. However, it may also be an indication of the microfibril formation from xylan-cellulose interaction. Furthermore, this lignin-xylan concentration trend for decomposition onset temperature is also observed for *T*_ΔMax_, which is the temperature at which decomposition occurs most rapidly. The results in Table 2 indicates that the initial addition of lignin lowers *T*_ΔMax_ (17% lignin = 261.1 °C) but then is followed by gradual increases in *T*_ΔMax_ values as a function of increasing lignin, values which approach that of 100% cellulose (278.1 °C). One explanation for this trend seen for both decomposition temperature and *T*_ΔMax_ is that the changing microfibril structures of the films alter according the relative lignin-xylan concentrations. When films contain higher xylan concentration, there are more associations between xylan and cellulose, affording more opportunity to produce microfibrils. However, without enough surrounding support of lignin due to low content, these xylan-cellulose microfibrils are not stable and can be more easily disrupted, hence the initial drop in temperature. As lignin concentrations increase, the xylan content is reduced and the lignin seems to exert a thermal resistance due to its interaction with the microfibrils. Consequently, the high lignin-concentrated films will allow the films to slightly resist decomposition at higher temperatures until a compositional event changes the structure, such as the change between 27.7% and 38% film. In this case, the morphology changes from fibrous to spherical. However, the onset temperature difference is negligible, suggesting a morphological alteration. This hypothesis is further supported by the observations of microfibril alterations and the formation of spherical domains in SEM, AFM, and X-ray scattering data analyses at micro- and nano-levels, respectively.

## 3. Materials and Methods

### 3.1. Materials

For the regenerated biomass films Avicel microcrystalline cellulose (Techware: Z26578-0) was purchased from Analtech (Newark, DE, USA). Xylan from beechwood (CAS: 9014-63-5) and lignin, alkali (CAS: 8068-05-1) were purchased from Sigma Aldrich (St. Louis, MO, USA). The ionic liquid used, 1-allyl-3-methylimidazolium chloride, was chosen due to its strong solvent abilities with cellulose, as demonstrated in past studies [30]. The 1-allyl-3-methylimidazolium chloride 98% (H26952) was purchased for Alfa Aessar (Haverhill, MA, USA).

### 3.2. Film Regeneration

The preparation of the regenerated biomass films was a modified version of the protocol seen in previous literature [28,53,54]. The appropriate amount of each biomass component was measured and then added together before its addition to the AMIMCl. The AMIMCl was then heated to about 85 °C, and then biomass components were added. The solution was dissolved for 30 min with stirring. The biomass solution was then spread evenly between two glass plates and fully submerged in distilled water for three ten-minute soakings, with a 2 min air drying interval in between soakings. After the third submersion, the plates were separated and individually submerged in distilled water. After ten minutes, the plates and the films were placed under N_2_ gas for ten minutes. Once this was completed the films were placed in a vacuum desiccator for 48 h for drying. For testing, the films were cut into pieces with dimensions of 2 mm square and 0.5 mm thick.

### 3.3. Attenuated Total Reflectance Fourier Transform Infrared (ATR-FTIR)

Attenuated total reflectance Fourier transform infrared (ATR-FTIR) analysis was performed using a Bruker ALPHA-Platinum FT-IR spectrometer with a platinum-diamond sample module and Bruker OPUS Mentor Plus software version 7.2 Build: 7.2.139.1294. The ATR-FTIR had settings of 128 background scans, 32 sample scans with a 4 cm^−1^ resolution, a 400–4000 cm^−1^ range, and 10% sensitivity for peak significance. For each sample, five spectra were taken at various locations on the film and an average spectrum was produced from these spectra, with this average used in the results.

### 3.4. Scanning Electron Microscope (SEM) and Atomic Force Microscopy (AFM)

Topological images from this study were taken with the LEO1450EP SEM at Rutgers University in Camden (NJ, USA). Samples prepared for analysis in the scanning electron microscope (SEM) were placed on a stub secured with a piece of double sided carbon tape. After placing the sample on the stub, the sample was coated with platinum or gold, layering until it reached a thickness of a few Angstroms. The EHT (extra high voltage/high voltage setting) was kept at 10.00 kV and the aperture size used was 30.00 µm. All images were captured at a magnification of 5000× amplification. An image included in this paper for Experiment 4 (38% lignin) was measured at a closer magnification (100,000×) to verify that the topological change from fibrous to spherical structures did in fact occur and can be seen in greater detail.

Atomic force microscopy scans were gathered on a Bruker Multimode 8 AFM at Rutgers University Camden campus. The samples of 2 mm square and 0.5 mm thickness were placed on the observation stub, secured using carbon tape, and placed on the observation stage. This was done in order to ensure that the preparation would not impact the topography of the sample. The parameters for AFM were a 10.0 µm scan size, 0.977 Hz scan rate, and drive amplitude of 100.00 mV.

### 3.5. X-ray Scattering

X-ray scattering was performed with the multi-angle X-ray scattering system (MAXS) at the University of Pennsylvania (Philadelphia, PA, USA). The MAXS system generates Cu-K X-rays, λ = 0.154 nm, from a Nonius FR 591 rotating anode operated at 40 kV and 85 mA. The bright, highly-collimated beam was obtained via Osmic Max-Flux optics and pinhole collimation in an integral vacuum system. The scattering data was collected using a Bruker Hi Star two-dimensional detector with sample-to-detector distances of 11, 54, and 150 cm, under vacuum. Each sample (standing film) had a thickness of approximately 0.5 mm. Pure powder samples were inserted into 1 mm glass capillary. All samples were dried in a vacuum for 24 h, before X-ray collection. The intensity reported is not absolute intensity and is, thus, reported in arbitrary units (a.u.). The X-ray scattering profiles were evaluated using Datasqueeze software; the isotropic 2-D scattering patterns were azimuthally integrated to yield an intensity versus scattering vector. For pure samples, the background was subtracted from an empty 1 mm glass capillary.

### 3.6. Thermogravimetric Analysis (TGA)

Thermogravimetric analysis (TGA) was conducted in a TA Discovery system, performed on all samples in a nitrogen atmosphere. Samples averaging 8 mg were heated linearly with a 10 °C/min ramp up to 650 °C. Step transition analyses were performed for each sample to determine the onset of decomposition (*T*_95%_) and the weight-loss percentage of the sample evolved during the main decomposition step. Derivative values were calculated and peak height analyses were performed to determine the temperature at which the samples decomposed at the fastest rates (*T*_ΔMax_).

## 4. Conclusions

In this study, we prepared regenerated films made from varying proportions of xylan, cellulose, and lignin using the ionic liquid AMIMCl. We observed variations in the structural, morphological, and thermal properties of the blended film as a function of component concentration. For example, in the FTIR-ATR analysis, the spectra showed increased peak similarity and intermolecular bonding changes according to component concentration. The findings on SEM showed significant differences in morphology, transitioning from fibrous to spherical surface structures with increasing lignin content. This was confirmed by AFM. These topographical alterations indicate changes to the intermolecular bonding of the films, representative of increased lignin wrapping around the microfibril structures up to a specific point, which exerts alterations to the morphology. The X-ray scattering data indicates a similar shift, at the nanoscale level, as a function of lignin-xylan concentrations. Xylan interacts with cellulose via hydrogen bonds resulting in an increase in the microfibril diameter. As the lignin content increases while reducing the xylan content, the microfibril diameter decreases and lignin self-assembled into spherical domains. The blended films formed intermediated semi-crystalline or amorphous structures depending on the constituent content. The thermal data acquired from TGA corroborates this trend, with the thermal stability increasing as a function of lignin and the disruption at 38% related to the change in the structure. From these findings, we suggest that the lignin-xylan concentration alters cellulose microfibril development in regenerated films, with higher xylan concentrations fostering larger microfibril diameters and lignin providing structural support to the xylan-cellulose microfibril structure. With the support and protection provided by lignin, the xylan-cellulose can more readily resist decomposition until a compositional alteration occurs causing the microfibril diameter to be reduce to a critical point. At this point, lignin seems unable to wrap around the microfibril and prefers to self-assemble into a spherical structure.

This study provides insight into possible avenues of regenerated film research, for the changes in lignin-xylan concentrations that can be exploited to change material properties to correspond with the demands and the utilization of natural materials as added-value products. More research investigating the relationship between these components during regeneration and their interaction should be pursued in order to understand how the microfibril diameter alters the material’s structural and physical properties. Particularly, research focusing on expanding the morphological characterization of blended polymer films would be especially useful to better understand the self-assembly of lignin in solid films. Thorough thermal characterization and computer simulations would elucidate in greater detail the molecular conformation of the microfibril structure and lignin, shedding further light on the morphology of this type of blended system.

## Figures and Tables

**Table 1:** Experimental conditions for regenerated films with a non-variable cellulose and variable lignin and xylan concentrations.

Relative Concentration of Lignocellulose				
Experiment Number	1	2	3	4
Microcrystalline Cellulose	100%	45%	45%	45%
Lignin	0%	17%	27.50%	38%
Xylan	0%	38%	27.50%	17%

**Table 2:** TGA decomposition temperatures of pure material components and experimental results.

Sample	*T*_Onset_ (°C)	*T*_End_ (°C)	Wt Loss (%)	*T*_ΔMax_ (°C)	Maximum Rate Wt Loss (%/°C)
Microcrystalline Cellulose	341.1	374.6	86.8	361.4	2.3%
Xylan	221.8	458.5	89.2	451.0	1.6%
Lignin	267.7	435.2	33.2	320.0	0.21%
100% Cellulose Film	249.7	300.4	77.8	278.1	1.4%
17% Lignin Film	228.1	293.8	75.9	261.1	1.0%
27.5% Lignin Film	235.5	289.1	73.7	263.5	1.2%
38% Lignin Film	235.7	286.9	74.2	266.5	1.3%
